# A critical appraisal tool for interdisciplinary study designs: Conceptual grounding and application in healthy ageing research

**DOI:** 10.1016/j.mex.2026.103830

**Published:** 2026-02-16

**Authors:** Andreea Alexandra Piriu, Maria Vittoria Bufali, Giulia Cappellaro, Amelia Compagni, Aleksandra Torbica

**Affiliations:** a Bocconi University, Via Roentgen 1, 20136 Milan, Italy; b Department of Social and Political Sciences, Bocconi University, Via Roentgen 1, 20136 Milan, Italy; c Centre for Research on Health and Social Care Management (CERGAS), SDA Bocconi School of Management, Via Sarfatti 10, 20136 Milan, Italy

**Keywords:** Public health, Healthy ageing, Interdisciplinary methodology, Critical appraisal, Quality assessment, Heterogeneous study design, Multidimensional research evaluation, Mixed-methods research, Intrinsic capacity, Functional ability

## Abstract

Interdisciplinary research often employs heterogeneous study designs that cannot be consistently addressed by existing quality appraisal frameworks. Established tools (CASP, JBI, EPHPP, STROBE) provide robust criteria, but no single framework adequately captures the methodological diversity typical of interdisciplinary work. Originating in a systematic review of 55 empirical studies on healthy ageing (Piriu et al., 2025), this article introduces the Nine-Item Quality Appraisal Toolkit (9-QuAT), a methodological innovation for critically assessing quality across diverse interdisciplinary study designs. The toolkit incorporates criteria from established frameworks, ensuring conceptual grounding, and extends them to include dimensions such as integration of methods, sample representativeness, and contribution to knowledge. Each criterion is operationalized in a binary scoring system, providing a structured yet flexible approach that enables reproducible and transparent assessment across heterogeneous designs, including mixed-methods, observational, descriptive, and exploratory studies. In doing so, the tool offers both a multidimensional research evaluation method and a transferable framework for interdisciplinary health and social research where traditional appraisal tools fall short. The article contributes along the following lines:

• Introduces a nine-item binary scoring toolkit for appraising interdisciplinary study designs.
• Extends established appraisal frameworks to capture interdisciplinarity and translational relevance.
• Demonstrates reliability, conceptual validity, and transferability across domains.

## Graphical abstract

**Figure:**
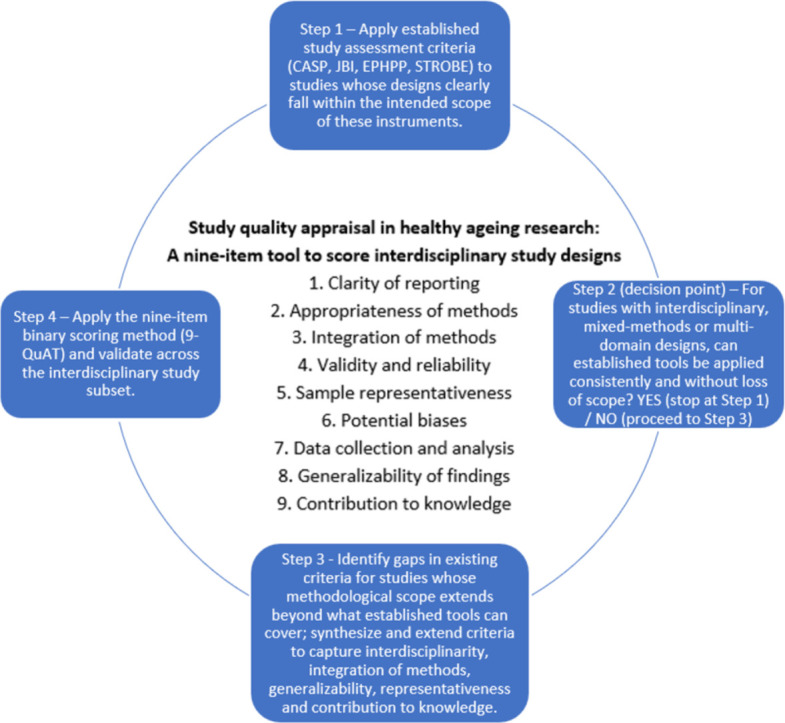

## Specifications table

**Table:** 

**Subject area**	Medicine and Dentistry
**More specific subject area**	Public Health; Healthy Ageing; Interdisciplinary Methodology
**Name of your method**	Nine-Item Quality Appraisal Toolkit (9-QuAT)
**Name and reference of original method**	N.A.
**Resource availability**	All resources are fully described in this article. Additional details are available in the following related resources: Systematic Review: https://doi.org/10.1016/j.socscimed.2025.117933 Data Article: https://doi.org/10.1016/j.dib.2025.111916 Dataset: https://www.doi.org/10.17632/gmvjxhdw2n.1

## Background

Healthy ageing (HA) is a multidimensional construct shaped by intrinsic capacity, functional ability, and environmental factors across the life course. Research in this field is increasingly interdisciplinary, spanning biomedical, psychosocial, and environmental sciences, with complex constructs operationalized through diverse measures and data sources. However, study designs associated with interdisciplinary research pose challenges for systematic appraisal. Existing tools provide robust criteria for specific designs, but no single framework adequately addresses the methodological heterogeneity typical of interdisciplinary research.

Building on the systematic review in Piriu et al. (2025a) [1], this article addresses the need for a critical appraisal tool tailored to interdisciplinary study designs and their operationalization. In response, it advances a methodological innovation – the Nine-Item Quality Appraisal Toolkit (9-QuAT). As shown in the review, the operationalization of HA is highly heterogeneous and lacks methodological comparability across the domains identified by the World Health Organization (WHO): intrinsic capacity, functional ability and environment. Furthermore, the companion PIETHA framework and dataset [2,3] document the specific measurement tools employed in HA operationalization, including specific measures, metrics, assessment scales, validated instruments and measurement methodologies used in practice. Developed during the appraisal of 55 empirical HA studies [1], 9-QuAT integrates and extends existing frameworks such as CASP [5], EPHPP [7], JBI [10] and STROBE [13], providing a structured, reproducible, and comprehensive approach to assessing methodological quality across heterogeneous designs.

Comparable efforts exist, such as the Quality Assessment with Diverse Studies (QuADS) tool [8], which was designed for mixed- and multi-method health services research. However, QuADS primarily emphasizes reporting quality and methodological transparency, while inviting a rather siloed approach. 9-QuAT differs by explicitly embedding integration of methods, sample representativeness and contribution to knowledge as transdisciplinary quality dimensions particularly salient in multidimensional HA research. In this way, the toolkit fills a methodological gap while also contributing to the broader literature on evaluating interdisciplinarity in health research.

The scoring method presented here not only complements the systematic review in identifying conceptual and measurement gaps, but also provides a structured means of evaluating how well interdisciplinary studies – particularly multi-domain HA research addressing environmental and psychosocial factors – are conducted and reported. These domains, as shown in Piriu et al. (2025) [1], remain comparatively neglected, making rigorous appraisal especially important. Importantly, 9-QuAT is not proposed as a replacement for established appraisal frameworks; rather, it provides a bridging structure when heterogeneous or multi-domain designs prevent consistent application of discipline-specific tools. The persistent inconsistency in critically appraising such heterogeneous designs is likely linked to the relative neglect of these domains. Therefore, by enabling more consistent and transparent appraisal, the toolkit not only strengthens the evidence base for healthy ageing but also steers future research toward a more balanced multi-domain exploration of biopsychosocial aspects of ageing. To operationalize this contribution, the following section details the development, structure and validation of 9-QuAT.

## Method details

9-QuAT is intended for interdisciplinary, mixed-methods, descriptive, exploratory, and multi-domain studies, as well as for reviews that synthesize heterogeneous evidence across domains. In these contexts, established appraisal tools (CASP, JBI, EPHPP, STROBE) cannot always be applied consistently and/or without loss of scope. 9-QuAT therefore provides a unified appraisal structure for such studies and can be complemented, where relevant, by discipline-specific checklists applied to individual components. Overall, the tool helps guide users in selecting 9-QuAT alone or in combination with existing tools. As outlined by the logic in the graphical abstract, for discipline-specific study designs of various types, established tools remain appropriate.

The toolkit was developed through a multi-step process. First, we systematically mapped quality assessment criteria from established frameworks: the CASP checklist for qualitative studies [5], the EPHPP tool for quantitative studies [7], the JBI checklists, especially the checklist for systematic reviews [10], and the STROBE guidelines for observational research [13]. Second, we applied these criteria to the 55 studies included in our systematic review of healthy ageing research, documenting areas of overlap, divergence and insufficiency. This exercise revealed a conspicuous gap: no single tool could adequately evaluate the methodological quality of interdisciplinary studies that were mixed-methods, observational, descriptive, or exploratory in nature – designs that frequently combine qualitative and quantitative elements and are increasingly common in healthy ageing research.

To address this gap, we examined the full 55-study sample in the systematic review in [1] and identified a subset of studies whose designs could not be appraised with existing tools. For this subset, we developed 9-QuAT by synthesizing criteria from existing tools through a structured comparison of their domains and the methodological features of these heterogeneous studies, and subsequently extended them to capture interdisciplinary features (see Table 1 below). We then applied 9-QuAT to this subset. The resulting tool comprises nine criteria: (1) clarity of reporting, (2) appropriateness of methods, (3) integration of methods, (4) validity and reliability of measures, (5) sample representativeness, (6) consideration of biases, (7) transparency of data collection and analysis, (8) generalizability of findings, and (9) contribution to knowledge. Each criterion is scored on a binary scale (0 = absent/poor, 1 = present/adequate), yielding a total score between 0 and 9. The binary scoring system was deliberately chosen to maximize reproducibility, comparability, and ease of application across interdisciplinary heterogeneous designs.

**Table 1:** 9-QuAT quality appraisal criteria for interdisciplinary study designs.

**1. Clarity of reporting**	No description or unclear description of methods and results: 0
	Clear description of methods and results: 1
**2. Appropriateness of methods**	Methods are not appropriate or poorly justified: 0
	Methods are appropriate and well justified: 1
**3. Integration of methods**	Lack of explanation or integration between qualitative and quantitative components: 0
	Clear explanation of how qualitative and quantitative data were integrated to provide a holistic understanding: 1
**4. Validity and reliability**	No assessment of validity and reliability conducted: 0
	Assessment of validity and reliability conducted for both qualitative and quantitative components: 1
**5. Sample representativeness**	Limited description of sampling strategies and lack of diversity in sample: 0
	Detailed description of sampling strategies and consideration of sample diversity: 1
**6. Potential biases**	Potential biases are not addressed or acknowledged: 0
	Potential biases are addressed or acknowledged: 1
**7. Data collection and analysis**	Lack of detail or transparency in data collection and analysis procedures: 0
	Detailed and transparent data collection and analysis procedures: 1
**8. Generalizability of findings**	Findings are not generalizable or applicability is not discussed: 0
	Findings are generalizable or applicability is discussed beyond the study context: 1
**9. Contribution to knowledge**	Limited contribution to knowledge or significance of findings not discussed: 0
	Significant contribution to knowledge or significance of findings discussed: 1

Each criterion is conceptually grounded in established appraisal traditions but tailored to interdisciplinary contexts. For example, “clarity of reporting” draws on STROBE, “validity and reliability” on EPHPP, and “contribution to knowledge” on CASP. Novel dimensions such as “integration of methods” explicitly operationalize interdisciplinarity by requiring evidence of how qualitative and quantitative strands are combined to generate a holistic understanding. Similarly, “generalizability of findings” and “sample representativeness” extend beyond traditional checklists to capture external validity and inclusivity, both critical in ageing research.

Table 2 provides a concise operational overview of the nine appraisal criteria used in 9-QuAT, with definitions and a rater guide detailing the scoring rules that specify what qualifies for the binary scores presented in Table 1. Binary scoring was chosen to minimize interpretational variability across heterogeneous designs. Scoring relied solely on published information. Absence of evidence was treated as evidence of absence (score 0). Borderline cases were resolved using rule-based operational discriminants: scores 1 only when explicit evidence was present in the study.

**Table 2:** Operational summary of 9-QuAT criteria and scoring rules.

Criterion	Operational definition	Borderline handling discriminants
**1. Clarity of reporting**	*Transparency of reporting of study design, methods and results* Methods and results are described and logically structured. Key methodological steps are present and adequately clear. Results are reported with description of how they were produced. The level of depth of the evidence presented allows reproducibility.	If reporting is concise but sufficient to allow reproducibility (i.e. design, methods and results are at least adequately, if not clearly, documented) → score 1 If key methodological steps are missing or ambiguous or if results are reported without evidence of how they were produced → score 0
**2. Appropriateness of methods**	*Alignment between aims, research question and methodological approach* Methods are justified and match research aim. The choice of methodological approach is clearly or adequately explained. The study considers if the goal of the research is discussed in relation to a justification of the research design e.g. has the researcher discussed how they decided which method to use?	For clear or adequate/implicit alignment → score 1 If methods appear inconsistent with research question or no rationale is provided → score 0
**3. Integration of methods**	*Analytical linkage between qualitative andquantitative or multi-domain components* Study explains how different data sources or methods inform each other. Integration between qualitative/quantitative or multi-domain elements occurs at interpretation, analysis or design level. Synthesis of methods is present and integration is analytically linked, with adequate explanation of cross-method contribution.	If there is adequate evidence of analytical linkage → score 1 Parallel reporting without synthesis → score 0
**4. Validity and reliability**	*Evidence of methodological rigour appropriate to study design* Trustworthiness, validation, or robustness are discussed. The measurement tools are defined, valid and reliable. Confounding variables or design limitations are credibly discussed. Reliability, robustness or validation statistics are provided for quantitative components. There is evidence of credibility, triangulation or reflexivity for qualitative components.	If there is adequate evidence of methodological rigour → score 1 If there is insufficient explanation or claims of robustness not supported by evidence → 0
**5. Sample representativeness**	*Transparency of sampling strategy andconsideration of diversity or scope* Sampling criteria and diversity/scope are addressed. Study discusses representativeness, diversity, or coverage. The sample selection is reported and adequately clear, with mentions of the target population or if/how probability sampling was attempted. Sample size justification, power description, or variance and effect estimates are preferably provided where appropriate.	If there is adequate evidence of sample selection, with justification → score 1 Small or purposive sample still score 1 if adequately justified, otherwise score 0.
**6. Potential biases**	*Acknowledgement of methodological or contextual limitations* Study explicitly discusses sources of bias (selection, measurement, reporting, etc.). Discussion of limitations must be methodologically and/or contextually relevant.	If there is evidence that discusses limitations are methodologically and/or contextually relevant → score 1 Generic statements only → score 0
**7. Data collection and analysis**	*Transparency of procedures used to collect and analyze data* Data collection methods are described (e.g. surveys, interviews, datasets). Analytical steps are adequately outlined and allow understanding of workflow. The overall analytical process must be supported by evidence.	If the analytical process is supported by evidence → score 1. Unclear or purely descriptive analytical process only → score 0
**8. Generalizability of findings**	*Consideration of applicability beyond the immediate study context* Study discusses transferability, scalability, policy relevance, or broader implications i.e. whether and how findings can be transferred to other populations or it considers other ways the research may be used. Findings are accompanied by contextual interpretation.	If there is evidence that the discussion is attempted with authenticity, reflexivity and at least an adequate level of depth → score 1 Qualitative studies may score 1 when discussing transferability rather than statistical generalization, otherwise score 0.
**9. Contribution to knowledge**	*Articulation of how findings advance knowledge andunderstanding, adding scientificvalue* Study identifies novelty, interdisciplinary insight, or synthesis contribution, discussing added value.	If authors discuss the study’s contribution to extant knowledge and understanding e.g. considering current practice/policy or relevant scientific literature, indicating future work areas → score 1 Policy commentary alone → score 0

Based on the 55-study sample in the systematic review (full list available in Table 1 in the Piriu et al. (2025) systematic review [1]), the 9-QuAT criteria were then applied to a heterogeneous subset of study designs (seven out of 55 studies) – specifically mixed-methods (e.g. [4,9]), observational cross-sectional surveys (e.g. [11,14]), descriptive or feasibility evaluations (e.g. [15,6]), and exploratory qualitative work [12]. While these designs differ in scope and methodology, they share the characteristic of being insufficiently covered by existing appraisal tools. Two reviewers independently scored each study against the nine criteria, with discrepancies resolved through consensus discussion. This process generated comparable quality profiles across diverse designs, demonstrating the toolkit’s pragmatic adaptability and its capacity to unify appraisal across non-RCT, interdisciplinary research. The 9-QuAT criteria and rater guide were iteratively developed during the quality assessment of studies included in the systematic review in [1] and subsequently applied to the heterogeneous study subset. The full operational definitions and scoring guidance, which were used throughout the appraisal process, are presented here in a more explicit and structured format to enhance transparency and reproducibility.

## Method validation

9-QuAT was designed for the appraisal of interdisciplinary, mixed-methods, and methodologically heterogeneous study designs that are not consistently covered by discipline-specific appraisal tools. Validation of 9-QuAT was embedded in its development and application during the systematic review process, including explicit operationalization of rating procedures, inter-rater reliability and sensitivity analyses.

### Differentiated use of established tools and the new toolkit

Following the logic presented in the graphical abstract, of the 55 studies included in the systematic review, 48 were appraised using established instruments (CASP for qualitative studies, JBI for systematic reviews, EPHPP for quantitative studies, and STROBE for observational designs). Seven studies with heterogeneous, multi-domain or mixed-methods designs ([4,6,9]), [11,12,14] and [15]) could not be consistently assessed using existing frameworks and were therefore appraised using 9-QuAT. This empirical distinction clarified the intended scope of 9-QuAT as a complementary tool designed to bridge methodological gaps rather than replace established appraisal instruments. Table 3 summarizes the distribution of quality scores across all study types.

**Table 3:** Quality assessment summary scores (Piriu et al., [1]).

Study type	Quantitative		Qualitative		Literature Review		Mixed-method, observational, descriptive, exploratory (9-QuAT)		Total	
	N. studies	% studies	N. studies	% studies	N. studies	% studies	N. studies	% studies	N. studies	% studies
High	28	68%	4	80%	1	50%	5	71%	38	69%
Moderate	13	32%	1	20%	1	50%	2	29%	17	31%
Low	0	0%	0	0%	0	0%	0	0%	0	0%
Total	41	100%	5	100%	2	100%	7	100%	55	100%

Cutoffs	Quantitative		Qualitative		Literature Review		Mixed-method, observational, descriptive, exploratory (9-QuAT)			
High	From 7 to 9		From 7 to 10		From 8 to 11		From 7 to 9			
Moderate	From 4 to 6		From 4 to 6		From 4 to 7		From 4 to 6			
Low	From 1 to 3		From 1 to 3		From 1 to 3		From 1 to 3			

### Reliability and construct validity

The 55 studies were independently scored by two reviewers, applying established appraisal tools or the 9-QuAT scoring (Table 1, Table 2) depending on the study design type. Inter-rater reliability was assessed during the systematic review phase. The inter-rater distribution of studies where established criteria could be applied was equal (24 studies/rater), and both raters appraised the quality of the subset of seven studies with diverse designs, subsequently comparing ratings and reaching raw agreement above 90%. Construct validity was established by mapping each of the nine criteria to recognized domains of methodological quality: for example, “clarity of reporting” aligns with STROBE, “validity and reliability” with EPHPP, and “contribution to knowledge” with CASP, demonstrating coherence with established appraisal frameworks.

As shown in Table 4, inter-rater reliability was evaluated using Cohen’s kappa (*κ*) calculated across all binary item ratings (7 studies x 9 criteria, 63 binary decisions) and across categorical quality levels in sensitivity analyses (high vs moderate, as per Table 3). For five items (CR, AM, DCA, GEN, CK), both raters assigned identical scores across all studies. These items showed perfect observed agreement but zero variance in both raters’ marginal distributions; therefore, Cohen’s kappa is mathematically undefined in these cases. For the remaining items, agreement beyond chance ranged from fair to perfect: IM (κ = 0.36), PB (κ = 0.72), VR (κ = 1.00) and SR (κ = 1.00), reflecting some expected judgement differences in heterogeneous mixed-methods appraisal. When all 63 binary decisions were pooled, the overall Cohen’s kappa was κ = 0.85, indicating substantial inter-rater reliability for the assessment tool as a whole.

**Table 4:** Independent ratings using 9-QuAT.

Study	Rater	CR	AM	IM	VR	SR	PB	DCA	GEN	CK	Total	Source of disagreement
Aung et al., 2022 [4]	R1	1	1	1	0	1	0	1	1	1	7	Full agreement
	R2	1	1	1	0	1	0	1	1	1	7	
Cyarto et al., 2013 [6]	R1	1	1	1	1	0	0	1	1	1	7	Integration item debated
	R2	1	1	0	1	0	0	1	1	1	6	
Howell et al., 2021 [9]	R1	1	1	1	1	0	1	1	1	1	8	Full agreement
	R2	1	1	1	1	0	1	1	1	1	8	
Moreno-Agostino et al., 2021 [11]	R1	1	1	1	1	1	1	1	1	1	9	Integration item debated
	R2	1	1	0	1	1	1	1	1	1	8	
Peterson et al., 2020 [12]	R1	1	1	0	0	0	1	1	1	1	6	Full agreement
	R2	1	1	0	0	0	1	1	1	1	6	
Wang et al., 2023 [14]	R1	1	1	1	1	0	1	1	1	1	8	Bias discussion interpreted differently
	R2	1	1	1	1	0	0	1	1	1	7	
Woo et al., 2021 [15]	R1	1	1	1	1	0	0	1	1	1	7	Full agreement
	R2	1	1	1	1	0	0	1	1	1	7	
Cohen’s kappa		N/A	N/A	0.36 (fair)	1.00 (perfect)	1.00 (perfect)	0.72 (substantial)	N/A	N/A	N/A		

Overall Cohen’s kappa: 0.85 (substantial reliability)

CR = Clarity of reporting, AM = Appropriateness of methods, IM = Integration of methods, VR = Validity and reliability, SR = Sample representativeness, PB = Potential bias discussion, DCA = Data collection and analysis, GEN = Generalizability, CK = Contribution to knowledge

After independent scoring, the assessors met to review all discrepancies. Each disagreement was discussed with reference to the operational definitions of the criteria, and a consensus rating was reached for every item. No third reviewer was required. Borderline cases – such as concise but sufficient reporting (CR), implicit but adequate methodological justification (AM), or small samples with clear rationale (SR) – were resolved by applying the rule that adequacy and evidential sufficiency warrant a score of 1, whereas ambiguity, missing methodological steps, or unsupported claims warrant a score of 0. This consensus procedure ensured that the final dataset reflected shared interpretation while preserving transparency regarding initial reliability.

### Sensitivity analysis

To address potential concerns regarding binary scoring, two sensitivity analyses were conducted: (1) conservative approach i.e. criteria were scored as present only when explicitly described in the study; (2) liberal approach i.e. implicit conceptual/methodological coherence was accepted as sufficient evidence. The analysis uses the operational discriminants in Table 2 to define which items are borderline and hence sensitive to interpretation, allowing to check if quality profiles remain stable under conservative and liberal scoring assumptions.

As presented in Table 5, four studies exhibited a one-point decrease and one study a two-point decrease in total score under conservative compared with liberal scoring, reflecting stricter interpretation of borderline-sensitive criteria (CR, AM, IM, SR, PB; notes in Table 5). Despite these shifts, we note that only two studies crossed the threshold between high and moderate quality when applying liberal vs. conservative scoring; notably, one of these (Cyarto et al., 2013 [6]) had already been classified as ‘moderate quality’ following borderline decision during the systematic review process. The remaining studies stayed within their original category (e.g. high-high or moderate-moderate). No other study changed quality category, and none shifted from high to low. This pattern, which is consistent with the item-level ratings in Table 4, indicates that 9-QuAT quality profiles remain stable under reasonable variation in interpretation and that borderline scoring does not materially alter study-level conclusions.

**Table 5:** Sensitivity of total scores (Liberal vs. Conservative Scoring).

Study	Liberal total (0–9)	Liberal profile	Conservative total (0–9)	Conservative profile	Profile Change (Liberal vs. Conservative profiles)
Aung et al., 2022 [4]	7	High	7	High	N
Cyarto et al., 2013 [6]	7	High	6	Moderate	Y
Howell et al., 2021 [9]	7	High	7	High	N
Moreno-Agostino et al., 2021 [11]	9	High	7	High	N
Peterson et al., 2020 [12]	6	Moderate	5	Moderate	N
Wang et al., 2023 [14]	8	High	7	High	N
Woo et al., 2021 [15]	7	High	6	Moderate	Y

CR = Clarity of reporting, AM = Appropriateness of methods, IM = Integration of methods, VR = Validity and reliability, SR = Sample representativeness, PB = Potential bias discussion, DCA = Data collection and analysis, GEN = Generalizability, CK = Contribution to knowledge

**Table:** 

CR	Concise but sufficient reporting:	Liberal: Adequate → 1 Conservative: Only if fully clear →1, otherwise concise/implicit → 0
AM	Implicit methodological justification	Liberal: implicit OK → 1 Conservative: Only explicit justification → 1
IM	Partial integration or limited explanation	Liberal: Some linkage → 1 Conservative: Only explicit analytical linkage → 1
SR	Small/purposive samples with rationale	Liberal: Justified → 1 Conservative: Only explicit justification → 1
PB	Partial or context‑light bias discussion	Liberal: Some relevant discussion → 1 Conservative: Only relevant methodological bias discussion → 1

### Added value and transferability

9-QuAT demonstrated added value by enabling structured and conceptually grounded appraisal framework for study designs that fall outside the scope of established discipline-specific tools. In doing so, it highlighted recurring strengths (e.g., clarity of reporting) and weaknesses (e.g., limited discussion of bias, insufficient integration of methods) across heterogeneous interdisciplinary studies. Its nine criteria draw on recognized methodological domains while explicitly extending them to capture interdisciplinary features such as integration of methods, sample representativeness, and contribution to knowledge. This makes the toolkit particularly suited to heterogeneous, multidomain, and mixed-methods studies, where traditional checklists cannot be applied consistently without loss of scope.

The worked example and sensitivity analysis presented in Table 4, Table 5 illustrate how item‑level ratings translate into total scores, and show that quality profiles remain stable across diverse designs and under reasonable rating variation. High-quality profiles signal consistently strong reporting and methodological alignment, whereas moderate profiles typically reflect specific limitations (e.g., partial integration of methods or limited discussion of bias) that warrant more cautious interpretation.

Finally, rather than replacing established tools, 9-QuAT functions as a pragmatic extension that complements frameworks such as QuADS by explicitly incorporating interdisciplinarity, translational relevance, and external applicability. Although developed in the context of healthy ageing, its criteria are sufficiently general to be applied to other interdisciplinary health domains, such as resilience, wellbeing, or social determinants of health. Its structured yet flexible design makes it adaptable to diverse research landscapes, while alignment with global initiatives underscores its potential for harmonizing interdisciplinary evidence.

### Limitations

Several limitations should be noted. First, the binary scoring system, while enhancing reproducibility, may oversimplify nuanced differences in study quality. Future iterations could explore weighted or Likert-scale scoring to capture gradations of quality. Second, the toolkit was validated on a relatively small sample of HA studies; broader application across disciplines is needed to confirm its robustness. Third, although the toolkit integrates criteria from established frameworks, it does not replace discipline-specific tools for purely qualitative, quantitative, or systematic review designs. Fourth, the toolkit has so far been applied only within the context of healthy ageing research, and broader application across other interdisciplinary health fields is needed to confirm its generalisability. Finally, as with all appraisal systems, the toolkit relies on the quality of reporting in the original studies, which may not always reflect underlying methodological rigour.

## Ethics statements

This study did not involve human participants, animals, or identifiable personal data. Ethical approval was therefore not required.

## Supplementary material

Additional information is available with the systematic review, the data article and the dataset in [1,2] and [3].

## CRediT authorship contribution statement

**Andreea Alexandra Piriu:** Writing – review & editing, Writing – original draft, Visualization, Validation, Supervision, Software, Resources, Project administration, Methodology, Investigation, Funding acquisition, Formal analysis, Data curation, Conceptualization. **Maria Vittoria Bufali:** Validation, Formal analysis, Conceptualization. **Giulia Cappellaro:** Validation, Conceptualization. **Amelia Compagni:** Validation, Project administration, Funding acquisition, Conceptualization. **Aleksandra Torbica:** Validation, Project administration, Funding acquisition, Conceptualization.

## Declaration of competing interest

The authors declare that they have no known competing financial interests or personal relationships that could have appeared to influence the work reported in this paper.

## Data Availability

No data was used for the research described in the article.
